# Brown Adipose Tissue and Skeletal Muscle ^18^F-FDG Activity After a Personalized Cold Exposure Is Not Associated With Cold-Induced Thermogenesis and Nutrient Oxidation Rates in Young Healthy Adults

**DOI:** 10.3389/fphys.2018.01577

**Published:** 2018-11-16

**Authors:** Guillermo Sanchez-Delgado, Borja Martinez-Tellez, Yolanda Garcia-Rivero, Juan M. A. Alcantara, Francisco M. Acosta, Francisco J. Amaro-Gahete, Jose M. Llamas-Elvira, Jonatan R. Ruiz

**Affiliations:** ^1^PROFITH (PROmoting FITness and Health through Physical Activity) Research Group, Department of Physical Education and Sports, Faculty of Sport Sciences, University of Granada, Granada, Spain; ^2^Department of Medicine, Division of Endocrinology and Einthoven Laboratory for Experimental Vascular Medicine, Leiden University Medical Center, Leiden, Netherlands; ^3^Nuclear Medicine Department, “Virgen de las Nieves” University Hospital, Granada, Spain; ^4^Nuclear Medicine Department, Biohealth Research Institute in Granada (ibs.GRANADA), Granada, Spain; ^5^Departament of Medical Physiology, School of Medicine, University of Granada, Granada, Spain

**Keywords:** brown fat, non-shivering thermogenesis, energy expenditure, energy balance, obesity, mild cold

## Abstract

Cold induced thermogenesis (CIT) in humans results mainly from the combination of both brown adipose tissue (BAT) and skeletal muscle thermogenic activity. The relative contribution of both tissues to CIT and to cold induced nutrient oxidation rates (CI-NUTox) remains, however, to be elucidated. We investigated the association of BAT and skeletal muscle activity after a personalized cold exposure with CIT and CI-NUTox in 57 healthy adults (23.0 ± 2.4 years old; 25.1 ± 4.6 kg/m^2^; 35 women). BAT and skeletal muscle (paracervical, sternocleidomastoid, scalene, longus colli, trapezius, parathoracic, supraspinatus, subscapular, deltoid, pectoralis major, and triceps brachii) metabolic activity were assessed by means of a ^18^Fluorodeoxyglucose positron emission tomography-computed tomography scan preceded by a personalized cold exposure. The cold exposure consisted in remaining in a mild cold room for 2 h at 19.5–20°C wearing a water perfused cooling vest set at 3.8°C above the individual shivering threshold. On a separate day, we estimated CIT and CI-NUTox by indirect calorimetry under fasting conditions for 1 h of personalized cold exposure. There was no association of BAT volume or activity with CIT or CI-NUTox (all *P* > 0.2). Similarly, the skeletal muscle metabolic activity was not associated either with CIT or CI-NUTox (all *P* > 0.2). The results persisted after controlling for sex, the time of the day, and the date when CIT was assessed. Our results suggest that human BAT activity and skeletal muscle ^18^F-FDG activity are not associated to CIT in young healthy adults. Inherent limitations of the available radiotracers for BAT detection and muscle activity quantification may explain why we failed to detect a physiologically plausible association.

## Introduction

Obesity is considered a public health problem of epidemic proportions (Ng et al., 2014). In simple terms, obesity results from a positive energy balance, and establishing a negative energy balance is a requisite for achieving weight loss. However, compensatory mechanisms, both physiological and behavioral, in response to short-term negative energy balance make it very difficult to establish a long-term energy deficit and sustainable weight loss (Palmer and Clegg, 2017). Thus, there are currently no non-invasive therapies capable of inducing sustainable weight loss, and developing new therapeutic strategies is, therefore, necessary (Palmer and Clegg, 2017).

Brown adipose tissue (BAT) was thought to be metabolically irrelevant or even absent in most human adults (Cannon and Nedergaard, 2004). However, recent evidence has shown that BAT is present and active in most, if not all, human adults (Nedergaard et al., 2007; Cypess et al., 2009; Saito et al., 2009; van Marken Lichtenbelt et al., 2009; Virtanen et al., 2009; Zingaretti et al., 2009). During the last decade, BAT has been regarded as a promising therapeutic target to tackle the obesity pandemic (Lee et al., 2013; Palmer and Clegg, 2017; Ruiz et al., 2018). Brown adipocytes are highly specialized thermogenic cells capable of taking up large quantities of energy substrates for producing heat by means of uncoupling mitochondrial respiration (Cannon and Nedergaard, 2004). In murine, BAT is responsible for a large proportion of both resting metabolic rate (RMR) and adaptive thermogenesis [i.e., diet-induced thermogenesis and cold-induced thermogenesis (CIT)] (Garland et al., 2011). However, although the total BAT volume in humans is still unknown (Martinez-Tellez et al., 2018), it is clear that adult humans present a considerable lower proportional amount (i.e., in relation to body weight) of BAT than murine (Leitner et al., 2017). Furthermore, murine and human BAT seem to have different molecular signatures and functionalities (Cypess et al., 2013; Muzik et al., 2013; Peirce et al., 2014). Therefore, it is still not clear whether human BAT is able to produce a relevant increase in the energy expenditure in adult humans (Marlatt et al., 2018).

The main reason why BAT contribution to human energy expenditure is still unknown is the lack of technology to properly assess its contribution *in vivo* (Ong et al., 2018). The most used technique to assess BAT volume and activity is the ^18^F-fluorodeoxyglucose (^18^F-FDG) positron-emission tomography and computed tomography (PET-CT) scan (Carpentier et al., 2018). Besides implicating high ionizing radiation exposure, one of the ^18^F-FDG-PET-CT scan’s main limitation relates to the substrate preference of BAT. The ^18^F-FDG radiotracer is a glucose analog. However, several studies have shown that brown adipocyte’s energy expenditure mainly relies on fatty acid oxidation (Schilperoort et al., 2016; Blondin et al., 2017). Although other alternatives to the ^18^F-FDG-PET-CT scan are being used, several limitations preclude the existence of a real gold-standard for *in vivo* BAT assessments in humans (Ong et al., 2018). Among the alternatives to ^18^F-FDG-PET-CT, the skin temperature of the supraclavicular area has been used as an indirect marker of BAT activity, which would allow non-invasive and continuous assessments (Boon et al., 2014; van der Lans et al., 2016).

Besides the technical limitations to study the BAT contribution to human energy expenditure, it has been suggested that BAT could just be a minor contributor to CIT in humans, while skeletal muscle, by means of both shivering and non-shivering thermogenesis, could be the main effector of CIT (Muzik et al., 2013; Blondin et al., 2015b; Jensen, 2015; U Din et al., 2016; Palmer and Clegg, 2017). Moreover, it has been suggested that not only skeletal muscle, but also white adipose tissue, could play a role in CIT (Blondin et al., 2015b; Betz and Enerbäck, 2017). To date, the are contradictory findings regarding the relative contribution of both human BAT and skeletal muscle to CIT (Muzik et al., 2013; van der Lans et al., 2013; Bakker et al., 2014; Chondronikola et al., 2014; Blondin et al., 2015b; Jensen, 2015; U Din et al., 2016; Yoneshiro et al., 2016; Palmer and Clegg, 2017; Porter, 2017), and more studies are needed to fully understand the relation of BAT and skeletal muscle activity with CIT. The relation of BAT and skeletal muscle activity with cold-induced nutrient oxidation rates (CI-NUTox) has received much less attention and remains to be elucidated. Changes in the pattern of nutrient oxidation are related to overall metabolic health (Galgani et al., 2008; Goodpaster and Sparks, 2017; Fernández-Verdejo et al., 2018). Thus, even if BAT or skeletal muscle non-shivering thermogenesis had a small impact on energy expenditure, they would still be very interesting therapeutic targets for human metabolic health improvements if they modify the substrate oxidation.

This study aimed to investigate the association of BAT and skeletal muscle ^18^F-FDG activity after a personalized cold exposure with CIT and CI-NUTox in young healthy adults. Additionally, we examined the association of supraclavicular skin temperature as a proxy of BAT activity with CIT and CI-NUTox rates.

## Materials and Methods

### Participants

We used data from two different cohorts. The participants were young (18–25 years old), healthy, did not smoke or take any medication, had had a stable body weight in the previous 3 months ( < 3 kg change), and were not regularly exposed to cold. A total of 57 young healthy adults (23.0 ± 2.4 years old; 25.1 ± 4.6 kg/m^2^; 35 women) participated in the present study (Table 1). Forty-four participants (29 women) were part of the ACTIBATE study (Study 1), a randomized controlled trial aiming to study the effect of exercise on BAT volume and activity (clinicaltrial.gov: NCT02365129) (Sanchez-Delgado et al., 2015). Only 18 out of these 44 participants met the required fasting time (6–8 h) to be included in the analyses referred to CI-NUTox (Table 1). The data for Study 1 was collected between October and November 2016. In addition, 13 participants were enrolled (Table 1) in Study 2, which was conducted between December 2017 and January 2018.

**Table 1:** Descriptive characteristics of the participants included in the energy expenditure analyses.

	CIT analyses (Study 1) (*n* = 44)	NUTox analyses (Study 1) (*n* = 18)	No PET-CT (Study 2) (*n* = 13)
Sex (women, %)	29 (65.9)	13 (72.2)	6 (46.2)
Age (years)	22.2 (2.2)	21.9 (2.0)	25.6 (3.0)
BMI (kg/m^2^)	25.6 (5.3)	24.3 (4.6)	23.6 (2.4)
Lean mass (kg)	42.7 (10.4)	40.4 (8.0)	45.7 (13.3)
Fat mass (kg)	27.2 (10.6)	25.0 (9.6)	18.4 (3.8)
Fat mass percentage (%)	37.0 (8.0)	36.1 (7.0)	28.4 (6.6)
RMR (kcal/day)	1565 (278)	1554 (227)	1484 (286)
BAT volume (ml)	94.4 (59.6)	74.29 (49.7)	
BAT SUV mean	4.29 (1.60)	4.24 (1.11)	
BAT SUV peak	14.13 (7.22)	13.40 (6.08)	
Muscle SUV peak	1.67 (0.33)	1.63 (0.33)	
Descending aorta SUV peak	0.92 (0.21)	0.83 (0.20)	


*Data are presented as means (standard deviation). BMI: Body mass index; RMR: Resting metabolic rate; BAT: Brown adipose tissue; SUV: Standardized uptake value; CIT: Cold-induced thermogenesis; NUTox: Nutrient oxidation rates.*

The participants signed a written informed consent, and both the informed consent and the whole study were approved by the Human Research Ethics Committee of the University of Granada (n°924) and of the Servicio Andaluz de Salud (Centro de Granada, CEI-Granada), and was performed following the Declaration of Helsinki (last revision).

### Procedures

Study 1. The data were collected on three days. The participants were always required to come to the research center by bus or by car (i.e., with the minimum possible physical activity), in a fasting state (>6 h), after having slept as usual, and having refrained from stimulant beverages and any moderate (within the previous 24 h) or vigorous (within the previous 48 h) physical activity.

On the first day, we assessed the participants’ shivering threshold (i.e., the lowest tolerable temperature without external observed or auto-reported shivering) (Martinez-Tellez et al., 2017c). After having checked that they met the previous conditions, the participants rested in a warm room for 30 min while wearing standardized clothes (Flip-flops, shorts, and a T-shirt; clo-value: 0.20). Later, the participants entered a mild cold room (19.5–20°C) and were equipped with a water-perfused cooling vest (Polar Products Inc., Ohio, United States) set at 16.6°C. They were required to remain seated and relaxed while the water temperature was progressively decreased (approximately 2°C every 10 min) until a temperature of 3.8°C was reached (at which the participants remained exposed for 45 additional minutes) or shivering occurred. We determined shivering visually and by asking the participants if they were experiencing shivering. The water temperature at which shivering occurred was considered the shivering threshold.

On the second day, we assessed BAT and skeletal muscle ^18^F-FDG activity by a static ^18^F-FDG PET-CT scan after a personalized cold exposure (Martinez-Tellez et al., 2017c). Prior to the PET-CT scan, the participants were exposed to a 2-h personalized cooling protocol, using the same water-perfused vest as in the shivering threshold test but set at 3.8°C above the individual shivering threshold, in a mild cold room (19.5–20°C). One hour after starting the cooling protocol, a bolus of approximately 5 mCi (≈185 MBq) of ^18^F-FDG was injected through a peripheral catheter, and the water temperature was increased by 1°C to avoid shivering. Immediately after the cooling protocol, we performed the static PET-CT scan and obtained PET-CT images from the atlas vertebrae (Cervical 1) to the thoracic vertebrae 6, approximately.

On the third day, we assessed CIT and CI-NUTox. After voiding their bladders, the participants wore the same standardized clothes (clo: 0.20) as on the other testing days and moved into a warm (23.2 ± 0.7°C) quiet room. Before the evaluation, the participants lay down on a reclined bed, in supine position, and covered by a sheet for 20 min. Later, RMR was assessed using indirect calorimetry for 30 min following the current methodological recommendations (Fullmer et al., 2015). They were instructed to breathe normally and not to talk, fidget, or sleep. After assessing RMR, the participants were moved into the cold room (19.5–20°C). They once again wore the temperature-controlled water perfused cooling vest set at the lowest tolerable temperature on the second day (i.e., 3.8°C above the individual’s shivering threshold, except for those who required changes in water temperature to avoid shivering during the cold-exposure previous to the PET-CT) (Study 1). Then, they lay down on a bed with the same reclined position as the one used for the RMR assessment and were instructed to breathe normally and not to talk, fidget, or sleep. Then, the CIT measurement was performed during two consecutive 30-min periods, separated by a 5-min pause to recalibrate the metabolic cart, during which they continued exposed to cold.

Additionally, on a different day, we measured the participant’s body composition by dual-energy x-ray absorptiometry scan (Discovery Wi, Hologic, Inc., Bedford, MA, United States). Weight and height were also measured by a Seca scale and a stadiometer (model 799, Electronic Column Scale, Hamburg, Germany).

Study 2. This study followed a similar procedure to Study 1, except for some minor differences. BAT and skeletal muscle ^18^F-FDG activity was not assessed, so Study 2 only included two testing days. On the shivering threshold test (day 1) the participants lay on a bed instead of being seated, and the fasting time before the CIT assessment was 10 h. Additionally, the time between the shivering threshold test and the CIT assessment was 48 h instead of 5–7 days.

### ^18^F-FDG-PET-CT Scan Analysis

We performed and analyzed the ^18^F-FDG-PET-CT scan (Siemens Biograph 16 PET-CT, Siemens, Germany) following the protocol extensively described elsewhere (Martinez-Tellez et al., 2017c, 2018) and in agreement with current methodological recommendations for human BAT assessment (Chen et al., 2016). We analyzed the images using the Beth Israel plugin for FIJI software (Schindelin et al., 2012). For the BAT assessment we applied a fixed range of Hounsfield units (HU, -190 to -10) (Chen et al., 2016) and an individualized SUV threshold: 1.2/(lean body mass/body mass) (Chen et al., 2016). We calculated BAT volume, BAT mean activity (SUV mean), and BAT maximal activity (SUV peak). In addition, we calculated the SUVpeak of several skeletal muscles (paracervical, sternocleidomastoid, scalene, longus colli, trapezius, parathoracic, supraspinatus, subscapular, deltoid, pectoralis major, and triceps brachii), and averaged the obtained value from all muscles in both sides of the body. Furthermore, we grouped these muscles into deep (paracervical, scalene, longus colli, paravertebral, subscapular), cervical (paracervical, sternocleidomastoid, scalene, longus colli), and cold sensitive (sternocleidomastoid, scalene, longus colli, pectoralis major) muscles, since it has been shown that these muscle groups could have a different behavior than others upon cold exposure (Blondin et al., 2015b). Additionally, a ROI was drawn in the descending aorta to be used as a reference tissue.

### CIT and CI-NUTox Estimations

The indirect calorimetry measurements for both RMR and CIT were performed using a neoprene face-mask connected to a CCM Express/Ultima CardiO2 metabolic cart (Medgraphics Cardiorespiratory Diagnostic, Saint-Paul, MN, United States) equipped with a directconnect^TM^ metabolic flow sensor (Medgraphics Corp, Minnesota, United States) (Sanchez-Delgado et al., 2017; Alcantara et al., 2018). The flow calibration was performed by a 3-L calibration syringe at the beginning of every test day, and the gas analyzers were calibrated using 2 standard gas concentrations before every 30-min bout of indirect calorimetry measurement following the manufacturers’ instructions. We used the same metabolic cart for RMR and CIT in all participants.

Indirect calorimetry data were averaged every minute and downloaded from the Breeze Suite 8.1.0.54 SP7 software (Medgraphics Cardiorespiratory Diagnostic, Saint-Paul, MN, United States). For RMR, we selected the most stable 5-min period (i.e., the one with the lowest average coefficients of variance of oxygen consumption, carbon dioxide production, minute ventilation, and respiratory exchange ratio), after excluding the first 5 min recorded (Sanchez-Delgado et al., 2017). To obtain a single representative value of CIT, we divided the 60 min recorded into 4 periods (i.e., 15 min each). We then selected the most stable 5-min period within every 15-min period (using the same criteria than for RMR). Finally, we used the 4 selected 5-min periods together with the RMR to calculate the area under the curve (trapezoidal rule), expressing it as a percentage of RMR (Sanchez-Delgado et al., 2018a).

Oxygen consumption and carbon dioxide production for each selected data point were used to estimate energy expenditure, carbohydrates (CHOox), and fat oxidation (FATox). Energy expenditure was estimated through Weir’s abbreviated equation (Weir, 1949). For CHOox and FATox estimations, we used Frayn’s equations (Frayn, 1983). We did not include urinary nitrogen data into the equations.

In addition to indirect calorimetry, we also recorded the skin temperature of several body locations (Martinez-Tellez et al., 2017a) throughout the CIT assessment by iButtons (DS-1922 L, Thermochron; resolution 0.0625°C; Maxim, Dallas, United States). All iButtons were attached to the skin with adhesive tape (Fixomull, Beiersdorf AG, Hamburg, Germany), and we estimated the mean skin temperature (ISO-standard 9886:2004, 2004). Finally, we calculated the difference between the warm value and the temperature for the subclavicular and supraclavicular skin temperature at the end of the cooling protocol. All data recorded by the devices were processed and analyzed by the Temperatus^®^ software^1^

### Statistical Analyses

The distribution of the variables was verified using the Shapiro–Wilk test, skewness and kurtosis values, visual check of histograms, Q-Q, and box plots. The descriptive statistics are presented as mean ± standard deviation, unless otherwise stated. The analyses were conducted using the Statistical Package for Social Sciences (SPSS, v. 21.0, IBM SPSS Statistics, IBM Corporation), and the level of significance was set at < 0.05.

We used simple linear regression analyses to test the association of BAT and skeletal muscle ^18^F-FDG activity after a personalized cold-exposure and supraclavicular temperature with CIT and CI-NUTox. We also used multiple linear regression models to test these associations adjusting by sex, BMI, the time of the day, and the date when CIT was assessed. Furthermore, we used repeated-measures analyses of variance (ANOVA) to study the cold-induced changes on skin temperature parameters. BAT and skeletal muscle ^18^F-FDG activity after a personalized cold-exposure was only assessed in Study 1. Therefore, Study 2 was only included in the analyses studying the association of the supraclavicular skin temperature with CIT and CI-NUTox.

## Results

**FIGURE 1:**
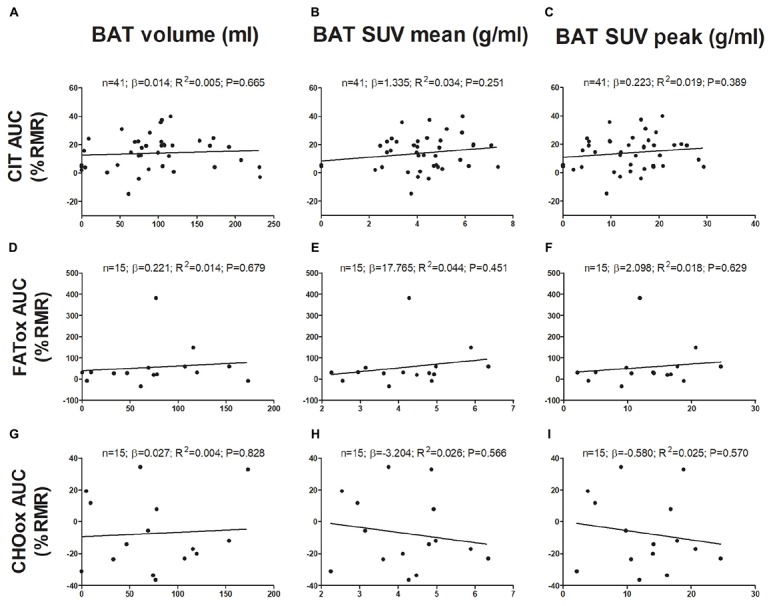
Associations of brown adipose tissue (BAT) ^18^F-FDG activity after a personalized cold exposure with cold induced thermogenesis (CIT) and cold-induced nutrient oxidation rates (Study 1). Unstandardized simple regression coefficient (β) and standardized coefficient of determination (R^2^). SUV: Standardized uptake value; AUC: Area under the curve; RMR: Resting metabolic rate; FATox: Fat oxidation rate; CHOox: Carbohydrates oxidation rate.

The associations of BAT with CIT and CI-NUTox are shown in Figure 1. There was no association of BAT (volume: all *P* > 0.68; mean activity: all *P* > 0.25; maximal activity: all *P* > 0.39) with CIT and NUTox. The results persisted after adjusting by sex, BMI, the time of the day, or the date when CIT was assessed. In addition, we analyzed whether using SUV expressed as a function of lean body mass (SUV_LBM_), instead of body mass (SUV_BM_), influenced the results (Leitner et al., 2017), and no differences were found (data not shown).

**FIGURE 2:**
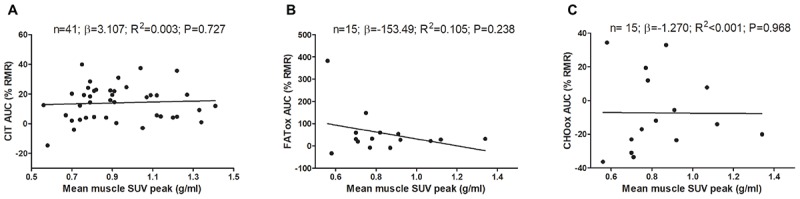
Association of skeletal muscle ^18^F-FDG activity after a personalized cold exposure with cold induced thermogenesis (CIT) and cold-induced nutrient oxidation rates (Study 1). Skeletal muscle ^18^F-FDG activity represents an average of the uptake in several skeletal muscles: paracervical muscles (cervical vertebrae 4), sternocleidomastoid, scalene, longus colli, trapezius, parathoracic muscles (Thoracic vertebrae 2), supraspinatus, subscapularis, deltoid, pectoralis major, and triceps brachii. Unstandardized simple regression coefficient (β) and standardized coefficient of determination (R^2^). SUV: Standardized uptake value; AUC: Area under the curve; RMR: Resting metabolic rate.

Figure 2 shows the association of skeletal muscle ^18^F-FDG activity after a personalized cold exposure with CIT and CI-NUTox. There were no associations either when using SUV_BM_ (all *P* > 0.23) or SUV_LBM_ (data not shown). Adjusting the analyses by sex, BMI, the time of the day, and the date when CIT was assessed did not modify the results. Furthermore, we tested the association of CIT and CI-NUTox with the deep, cervical, and cold sensitive muscles activity, as they have been shown to respond differently to cold (Blondin et al., 2015b; Martinez-Tellez et al., 2017c). We found no significant association with any criteria for grouping muscles (i.e., deep muscles, cervical muscles, and cold sensitive muscles). All these results remained when using skeletal muscle SUVmean instead of SUVpeak (data not shown).

**FIGURE 3:**
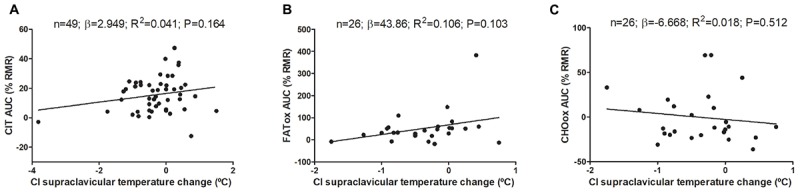
Association of cold-induced (CI) supraclavicular skin temperature change with cold induced thermogenesis (CIT) and cold-induced nutrient oxidation rates (including participants of studies 1 and 2). Unstandardized simple regression coefficient (β) and standardized coefficient of determination (R^2^). AUC: Area under the curve; RMR: resting metabolic rate.

Changes on mean, subclavicular, and supraclavicular skin temperature during CIT assessment are shown in Supplementary Figure S1. The associations of cold-induced changes in supraclavicular skin temperature with CIT and CI-NUTox are shown in Figure 3 (including data of studies 1 and 2). We failed to observe any significant association (all *P* > 0.09), which was unaffected when adjusting by sex, BMI, the time of the day, or the date when CIT was assessed. Similar results were found when using the skin temperature data at the end of the test instead of the cold-induced change (Δ). Moreover, neither the mean nor subclavicular skin temperature were associated with CIT or CI-NUTox.

Finally, we also tested the above-mentioned associations using the difference between energy expenditure at the end of the cooling protocol and RMR, instead of the area under the curve calculation, and with % of energy expenditure coming from FATox instead of CI-NUTox. We found no significant associations in any of these analyses (data not shown).

## Discussion

This study analyzed the association of BAT and skeletal muscle ^18^F-FDG activity after a personalized cold exposure with CIT and CI-NUTox in young healthy adults. We also examined the association of supraclavicular skin temperature, an indirect marker of BAT activity (Boon et al., 2014; van der Lans et al., 2016), with CIT and CI-NUTox. No significant associations were found of BAT, skeletal muscle ^18^F-FDG activity, or supraclavicular skin temperature with CIT and CI-NUTox. This lack of association was consistent across different methodologies for BAT and CIT assessment, and independent of several potential confounders. These findings are partially in line with other studies which used different methodologies (Muzik et al., 2013; Blondin et al., 2015b; U Din et al., 2016), suggesting a negligible contribution of BAT to human CIT. On the other hand, the observed associations of skeletal muscle ^18^F-FDG activity after a personalized cold exposure with CIT and CI-NUTox should be considered with caution, since not having ^18^F-FDG activity in warm conditions might impair the ability to effectively assess cold-induced skeletal muscle metabolism.

The relation between BAT and CIT in humans has been extensively studied during the last years, yet, controversial results still exist. Several studies showed that individuals with detectable BAT (BAT + in ^18^F-FDG-PET-CT scan) present higher CIT levels (Vijgen et al., 2011; Yoneshiro et al., 2011; Chondronikola et al., 2014), and that only BAT + individuals present seasonal variation of CIT, being higher in winter than in summer (Yoneshiro et al., 2016). Moreover, other studies showed positive and significant associations between BAT (assessed by ^18^F-FDG-PET-CT) and CIT (van Marken Lichtenbelt et al., 2009; Ouellet et al., 2012; Chen et al., 2013). In contrast, other studies did not observe any significant association between BAT and CIT (Vosselman et al., 2012; Bakker et al., 2014; Blondin et al., 2015b), and BAT activation induced by cold acclimation was not accompanied by changes in CIT (Lee et al., 2014), which concur with our findings. Of note is that the lack of association observed in our study between CIT and supraclavicular skin temperature, as an indirect marker of BAT activity (Boon et al., 2014; van der Lans et al., 2016), further reinforces this finding.

Importantly, studies using [(15O)O_2_], instead of ^18^F-FDG, as the radiotracer for PET-CT scans, have demonstrated that the direct contribution of BAT to CIT is rather small (i.e., only 1% of the increase of CIT) (Muzik et al., 2013; U Din et al., 2016). Of note is that although [(15O)O_2_] presents limitations due to a very short half-life (e.g., only a small anatomical area can be assessed) among others, it is able to effectively quantify energy expenditure of different tissues and it is not affected by changes in substrate preference, as ^18^F-FDG is. According to these studies, BAT in the cervical and upper thorax area (most human BAT) would account for only 10–15 kcal/day if fully activated for 24 h. However, paradoxically, some of the studies using radiotracers different from ^18^F-FDG consistently showed a positive association of BAT perfusion and volume with CIT (Orava et al., 2011; U Din et al., 2016). This, together with the observations showing higher glucose uptake and energy expenditure in skeletal muscles close to BAT depots (Blondin et al., 2015b; U Din et al., 2016), suggest that BAT may influence human CIT by indirect, rather than direct, mechanisms (U Din et al., 2016), such as endocrine signaling (Villarroya et al., 2017).

The hypothesis of an indirect effect of BAT over CIT could explain the controversy in the studies investigating the relation between BAT assessed by ^18^F-FDG-PET-CT and CIT. Moreover, it is known that different methodological approaches for both PET-CT acquisition and analysis can profoundly influence the outcome (Martinez-Tellez et al., 2017b, 2018). Most studies examining the relation between BAT and CIT were conducted before a consensus was reached on how to perform PET-CT scans for BAT assessment and quantification (Chen et al., 2016), and thus, applied different methodologies. Therefore, methodological issues regarding cold exposure prior to PET-CT and PET-CT analyses might explain the observed discrepancies. Here, we investigated the association between BAT and CIT in a larger sample size than previous studies, and strictly following state-of-the-art methodology for BAT assessment. However, it is to note that we measured BAT and CIT on different days, and, therefore, intra-individual day-to-day variance in energy expenditure may have prevented us from finding an existing association (Bader et al., 2005; Schoeller, 2007; Cooper et al., 2009; Alcantara et al., 2018).

There is cumulating evidence supporting the idea that skeletal muscle is the main thermogenic organ upon cold exposure in humans (Blondin et al., 2015b; Jensen, 2015; U Din et al., 2016; Betz and Enerbäck, 2017), even at mild cold exposure. For instance, upon cold stimulation, energy expenditure of muscles in the cervical and upper thorax is ≈8 times higher than energy expenditure of BAT (U Din et al., 2016). Interestingly, skeletal muscle contribution to CIT seems to be higher in deep and centrally located muscles than in superficial and bigger muscle groups (Blondin et al., 2015b; U Din et al., 2016). Moreover, it is not clear whether the muscle energy expenditure during mild cold exposure relies upon shivering (Blondin et al., 2015b) or non-shivering mechanisms (Betz and Enerbäck, 2017). In contrast with this strong evidence, we found no association between skeletal muscle ^18^F-FDG activity after a personalized cold exposure and CIT. However, it should be considered that we did not assess the skeletal muscle ^18^F-FDG activity in warm conditions, and, therefore, we could not determine whether the cold-induced change in glucose uptake was associated with CIT. This issue might not be of importance where BAT is concerned (Chen et al., 2016), since BAT glucose uptake in warm conditions is rather low (Cypess et al., 2009). However, differences between muscle ^18^F-FDG activity in warm conditions and upon cold exposure are much smaller, and, therefore, not having skeletal muscle warm ^18^F-FDG activity might have considerably limited the ability to detect an existing association. In addition to the skeletal muscle ^18^F-FDG activity, we also tested the association between lean mass (as a subrogate of muscle mass) and CIT, which did not show significance (data not shown).

Moreover, it should be noted that skeletal muscle thermogenesis, even during shivering, relies mainly on fatty acid oxidation (Blondin et al., 2014; Haman and Blondin, 2017), and, therefore, the glucose analog ^18^F-FDG might not be a valid marker of muscle thermogenesis or metabolic activity. Similarly, BAT thermogenesis also relies mainly on fat oxidation (Blondin et al., 2017), and it has recently been shown that glucose uptake is not mandatory for human BAT thermogenesis (Blondin et al., 2015a). Therefore, inherent limitations of ^18^F-FDG for BAT detection and muscle activity quantification may explain why we failed to detect a physiologically plausible association. There is a need to develop new radiotracers for BAT detection and muscle activity quantification with more metabolic significance than ^18^F-FDG and with a larger half-life than others such as [(15O)O_2_].

We also studied the associations of BAT and skeletal muscle ^18^F-FDG activity with CI-NUTox. Since both BAT and skeletal muscle thermogenesis relies on fatty acid oxidation, it is plausible to expect a positive association of BAT and skeletal muscle activity with FATox. In contrast, we observed no association, which could be partially explained by the inherent limitations of ^18^F-FDG as a radiotracer. However, since BAT and skeletal muscle thermogenesis seem to compensate each other (Blondin et al., 2016) and both mainly depend on FATox, it is also plausible that no relation with FATox exists. Finally, it should be considered that we recorded CI-NUTox in a cold exposure of only 1 h. Longer cold exposures result in different contributions of both CHOox and FATox (Blondin et al., 2014), and, therefore, new studies examining the relation of BAT and muscle thermogenesis with CI-NUTox during longer cold exposures are needed.

Our results should be considered with caution since some limitations are present. It should be noted that BAT and skeletal muscle ^18^F-FDG activity was assessed on a different day than CIT and CI-NUTox, and, therefore, day-to-day variation may have influenced our results. Moreover, as stated above, whereas ^18^F-FDG PET-CT after a personalized cold exposure is currently considered the gold standard for BAT *in vivo* quantification (Chen et al., 2016; Carpentier et al., 2018), it is not the best method to assess skeletal muscle metabolism upon cold exposure, which could explain the lack of association with CIT. Moreover, we quantified skeletal muscle ^18^F-FDG activity (SUVpeak) in one image slide, and therefore it might be influenced by the blood vessels eventually contained in the ROI. Using skeletal muscle SUVmean did not change the results, probably because muscle SUVmean and SUVpeak are highly correlated (all *r* > 0.976; all *P* < 0.001). Of note is also that the cooling protocol applied to assess both BAT and skeletal muscle ^18^F-FDG activity and CIT and CI-NUTox is based on the individuals’ shivering threshold, which was assessed by subjective methods (self-reported and direct observation), rather than by objective methods (electromyography) (Acosta et al., 2018). Another relevant issue is that the cold-exposure used to assess CIT and CI-NUTox was only 1 h long, and, therefore, we cannot know whether a longer cold exposure would provide different results. In addition, it should be noted that we studied young healthy adults, hence we do not know whether these findings extend to older or unhealthy individuals. Finally, due to a lack of homogeneity in the fasting time of Study 1, we conducted the NUTox analyses with a relatively small sample size (only 18 out of 44 participants in Study 1). However, we performed a second study which allowed us to study the association of supraclavicular temperature with CIT and CI-NUTox in a larger sample size.

## Conclusion

We found, in a larger sample size than previous studies and strictly following the most updated methodological recommendations, that BAT and skeletal muscle thermogenic activity (assessed by means of ^18^F-FDG activity after a personalized cold exposure) is not associated with CIT or CI-NUTox. These findings support the hypothesis of BAT having a marginal role in human CIT, although important limitations inherent to the available technology for BAT and skeletal muscle metabolism *in vivo* quantification precludes us from drawing firm conclusions from the present data.

## Author Contributions

GS-D, BM-T, and JR designed the research. GS-D, BM-T, YG-R, JA, FA, FA-G, and JL-M conducted the research. GS-D, BM-T, and JM analyzed the data. GS-D, wrote the paper. GS-D and JR had primary responsibility for the final content. GS-D, BM-T, YG-R, JA, FA, FA-G, JL-M, and JR, discussed the results and approved the final version of the manuscript.

## Conflict of Interest Statement

The authors declare that the research was conducted in the absence of any commercial or financial relationships that could be construed as a potential conflict of interest.

## Footnotes

1 http://profith.ugr.es/temperatus?lang=en

## Supplementary Material

FIGURE S1 Skin temperature parameters during cold-induced thermogenesis. *P* for one-way analysis of variance. min: minute.
