# Supplementary figures and images for: Brown Adipose Tissue and Skeletal Muscle 18F-FDG Activity After a Personalized Cold Exposure Is Not Associated With Cold-Induced Thermogenesis and Nutrient Oxidation Rates in Young Healthy Adults
Source: Front Physiol. 2018 Nov 16;9:1577. doi: 10.3389/fphys.2018.01577 (PMC6250802; doi:10.3389/fphys.2018.01577)

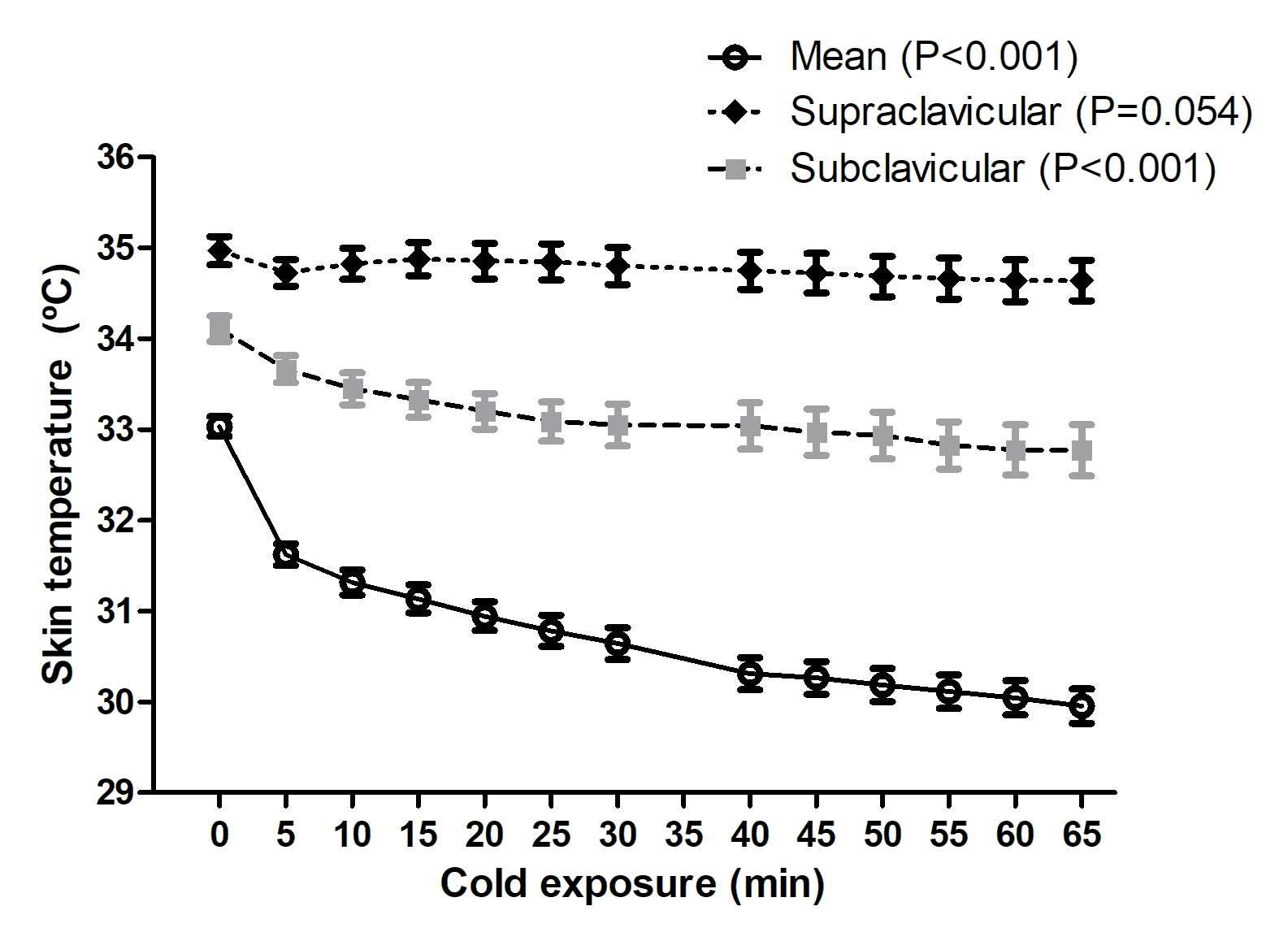

Supplement: FIGURE S1 — Skin temperature parameters during cold-induced thermogenesis. P for one-way analysis of variance. min: minute. [file Image_1.tif]
